# Beyond Barriers: Reconsidering Cultural Sensitivity in Hospice and Palliative Care Services by Applying the Empathy Map

**DOI:** 10.1007/s10903-025-01838-z

**Published:** 2025-12-23

**Authors:** Sabahat Ölcer, Annika Albert, Christian Banse, Friedemann Nauck, Maximiliane Jansky

**Affiliations:** 1 Department of Palliative Medicine, University Medical Center Göttingen, University of Göttingen, Göttingen, Germany; 2 Department of Human-Centered Technology Development, Institute of Computer Science, Ruhr West University of Applied Sciences, Bottrop, Germany

**Keywords:** Palliative care, Patients with a migration background, Empathy map, Healthcare providers, Cultural sensitivity

## Abstract

Culturally sensitive palliative care for patients with a migration background (PwM) remains a critical but underexplored field, especially in the German healthcare context. Our study employs Dave Gray’s empathy map to capture healthcare providers’ perspectives on the challenges, needs, and experiences of PwM in hospice and palliative care. We investigated palliative care challenges through open-ended questions in an online survey conducted across various hospice and palliative care settings. Based on 332 completed responses, our analysis revealed significant barriers to delivering culturally sensitive care. Using qualitative content analysis, we applied an empathy map to identify categories. Findings showed that language barriers and cultural insensitivity notably hindered communication, access, and trust-building. PwM often relied on family networks, which caused delayed care-seeking and logistical difficulties. Despite these challenges, PwM strived for dignity, trust, and care aligned with their cultural background. Our study highlights the importance of empathy-based approaches in hospice and palliative care and the effectiveness of the empathy map as an instrument for identifying challenges and gaps. By addressing the specific needs of PwM, the findings contribute meaningful insights for healthcare providers, policymakers, and researchers.

## Introduction

Palliative care for patients with a migration background (PwM) involves multiple challenges that impact the quality and accessibility of end-of-life services [1, 2]. Language barriers, cultural misunderstandings, and a lack of culturally sensitive care often prevent effective hospice and palliative care (HPC) for this population group [1, 3–7]. These challenges are further complicated by limited knowledge of HPC services, fear or distrust of healthcare systems, poor provider-beneficiary communication, and socioeconomic factors [1, 4, 8–10]. Furthermore, particularly in the context of immigration, individuals with traumatic experiences may show complex health problems that healthcare providers are currently unprepared to address [11, 12]. These barriers cause PwM to be unable to access care services that meet their cultural, spiritual, and emotional needs at the end-of-life [13].

Family members of PwM play a central role in informal care, but face significant difficulties. While culturally important, reliance on family care can lead to emotional and practical strain, particularly when family members lack the resources or knowledge to handle complex healthcare systems [9, 10]. PwM experience unmet needs, poor communication, and insufficient culturally appropriate support in HPC [4, 12]. A US-based study indicates that they are nearly twice as likely as the local population to receive burdensome, potentially unnecessary aggressive care in their final weeks [13]. Factors such as lower health literacy and lack of advance care planning documentation increase these disparities [14]. Addressing these issues requires culturally tailored training programs and community outreach strategies [8, 10] to ensure that PwM and their families receive the compassionate and high-quality HPC they deserve [3, 14].

Shabnam et al. [14] suggest developing complex interventions to support this population group and emphasise the importance of tailored approaches. To meet the specific needs of PwM in HPC, healthcare providers should consider sociocultural and emotional factors as well as physical symptoms. This includes understanding how cultural norms affect views on illness, death, dying, and caregiving roles within these communities [1, 3, 4, 13]. By considering their perspectives, healthcare providers can foster empathy and understand the emotional, cultural, and spiritual aspects of care that PwM need.

Empathy has emerged as a key element in healthcare, particularly in palliative care, where cultural sensitivity and emotions are crucial [15]. Tan et al. [16] define clinical empathy as a developing skill that requires healthcare providers to connect with patients’ emotional and cultural needs. Boucher and Johnson [17] emphasise the importance of teaching hospice staff in culturally sensitive empathy to address barriers faced by diverse patients. Recent studies have explored different ways to strengthen empathy in healthcare [16, 18]. For instance, Cairns et al. [19] demonstrated that empathy maps effectively improve medical students’ communication skills and deepen their understanding of patient perspectives.

Empathy mapping, developed by Dave Gray, is a qualitative tool used to visualise what people think, feel, say, do, see, hear, experience as pain, and seek as gain in a given context [20]. Beyond its educational use, we applied the empathy map as an analytical framework to better understand how empathy and cultural sensitivity manifest in real palliative care contexts, rather than as a training tool with healthcare providers. This also allowed us to explore how healthcare providers interpret and respond to PwM’s experiences, rather than teaching empathy as a concept.

We used these dimensions as a practical framework to capture the cultural, emotional, and social experiences of PwM in HPC settings in Germany. We aimed to derive insights that enable healthcare providers to better visualise and understand PwM’s specific needs, thereby supporting the development of culturally tailored communication and care strategies. Despite increasing diversity, research on the practical barriers in providing culturally sensitive palliative care to PwM remains scarce. Through this structured approach, we examined how healthcare providers perceive and interact with PwM, offering a focused perspective on the difficulties they encounter in delivering equitable care. This approach matches recent changes in healthcare education that emphasise hands-on learning and reflection to build empathy and cultural competence [17, 21]. By mapping these relationships, we aimed to identify potential targets that could inform future empathy training and contribute to more effective and culturally sensitive palliative care for PwM.

## Methods

The empathy map is a qualitative instrument designed to systematically capture and organise an individual’s or group’s perceptions, feelings, and experiences in a specific context [19, 20]. In this study, the empathy map was not used as a participatory tool with respondents but as an analytical framework to structure and interpret the qualitative data. We applied Dave Gray’s empathy map framework [20], which enables a detailed exploration of what PwM see, hear, think, feel, say, do, experience as pain and seek as gain, as interpreted by the healthcare providers. This framework guided the analysis rather than the data collection process, providing a clear structure for analysing the data using its predefined categories as a guide. Our study covered various HPC settings across Germany, with recruitment facilitated by the German Association for Palliative Medicine (*Deutsche Gesellschaft für Palliativmedizin*, DGP), which provided contact information for the facilities. We used an online survey with open-ended questions. Our study received approval from the Institutional Review Board of the University Medical Center Göttingen.

### Participants

We drew on a non-probability voluntary response sampling to reach healthcare providers in HPC facilities in Germany. Participants consisted of healthcare providers who provide HPC to individuals nearing the end of their lives. Our sample included 332 healthcare providers, described by demographic and professional characteristics. Most were female (76.5%), followed by males (23.2%) and a small representation of diverse identities (0.3%). Age ranges revealed the largest group between 51 and 60 years (50%), with smaller groups in other age categories. Professionally, coordinators constituted the largest group (36.1%), followed by physicians (24.7%) and nurses (21.4%). Psychologists (1.8%), social workers (8.4%), spiritual workers (0.6%), volunteers (1.2%), and others (5.7%) represented smaller proportions. The data showed that participants worked across various facility types, with multiple options possible: 44.3% were from volunteer hospice services, 28.9% specialised palliative homecare teams (SAPV), 18.1% palliative care units, 14.8% inpatient hospice services, 6.6% palliative care advisory teams in hospitals, and other facilities.

### Data Collection

We developed our study instrument by adapting and modifying an online survey previously used in a 2017 study focusing on HPC for PwM in Lower Saxony, Germany [22, 23]. The original survey contained 63 items, including 20 complex items that allowed conditional follow-up questions depending on participant responses. It covered: (1) information about the institution, (2) PwM in the facility and perceived access barriers, (3) case descriptions of the last cared for PwM and identified problems and solutions, (4) provision of care in the facility including migration-specific offers and cooperation with other institutions, (5) employees with migration backgrounds, (6) demographic data of participants, and (7) desired support in caring for PwM. The survey underwent a revision process to improve clarity, understandability, and usability. This included pretesting and pilot testing with research team members and healthcare professionals, adjustments based on feedback, ensuring technical functionality for online administration, and the correct operation of complex and conditional items. Two rounds of pretesting confirmed comprehension and response depth. AA prepared an initial revised version of the survey, which was then thoroughly reviewed and discussed by CB, MJ, and FN. Following this collaborative process, the finalised survey was used as our primary data collection tool. The survey included open-ended questions to explore healthcare providers’ experiences with PwM, addressing access barriers, care strategies, case descriptions, immigration-specific services, and cooperation between facilities.

AA obtained a table with the email addresses of almost all HPC facilities in Germany from the “*Wegweiser Hospiz- und Palliativversorgung* (Guide to Hospice and Palliative Care)”, an online directory maintained by the German Association for Palliative Medicine [24], which simplified our recruitment process. AA then contacted these facilities via email, asking them to forward the online survey link to eligible healthcare providers within their facilities. We utilised 2,711 listed adult care facilities to recruit potential study participants. For data collection, we employed open-source software developed by Arslan and Tata [25], which enables the design of questionnaires with varying complexity. We distributed the online survey on November 27, 2019, and it remained open until February 1, 2020. Following the exclusion of incomplete responses, our final analysis included 332 completed surveys. To ensure data quality, we included clear instructions, screened responses for completeness and consistency, and removed superficial or duplicate entries.

### Data Analysis

Our analytical approach comprised two components: directed qualitative content analysis, as described by Hsieh and Shannon [26], for responses to open-ended questions, and descriptive statistical analysis for demographic and professional characteristics of participants, and facility types. We chose the directed approach because our analysis was guided by an existing theoretical framework, the empathy map, which provided predefined categories and concepts. This approach allowed us to systematically interpret the data in relation to these categories while remaining open to new insights emerging from participants’ responses. Our study employed IBM SPSS Statistics (Version 26) and MAXQDA (Version 2020) for all data analysis. AA performed descriptive statistical analyses, including the assessment of frequencies and percentages for this study. For the qualitative part, we followed a deductive coding process guided by the structure of the empathy map framework developed by Dave Gray. We guided the text analysis by the dimensions of the empathy map: see, hear, think, feel, say, do, experience as pain, and seek as gain [20]. This framework provided a clear structure for analysing the data using predefined categories as a guide.

We began our analysis with a comprehensive review of all responses. Two researchers, AA and SÖ, independently completed the first review to become familiar with the data. Following this, AA and SÖ carried out the initial coding separately to avoid bias in data interpretation. After comparing all codes, we identified and resolved potential discrepancies, ensuring a robust and reliable process. Following the establishment of coding consistency, SÖ developed the categories and themes based on the dimensions of the empathy map. All authors engaged in collaborative discussions to compare, revise, and finalise codes, categories, and themes. Thus, we achieved consensus and maintained the reliability of our findings. Moreover, our study protocol included providing participants with detailed information about the study’s purpose. We obtained their consent before allowing them to proceed with the survey. To ensure anonymity, we used the abbreviation ‘Respondent (R)’ throughout the process.

## Results

**Fig. 1:**
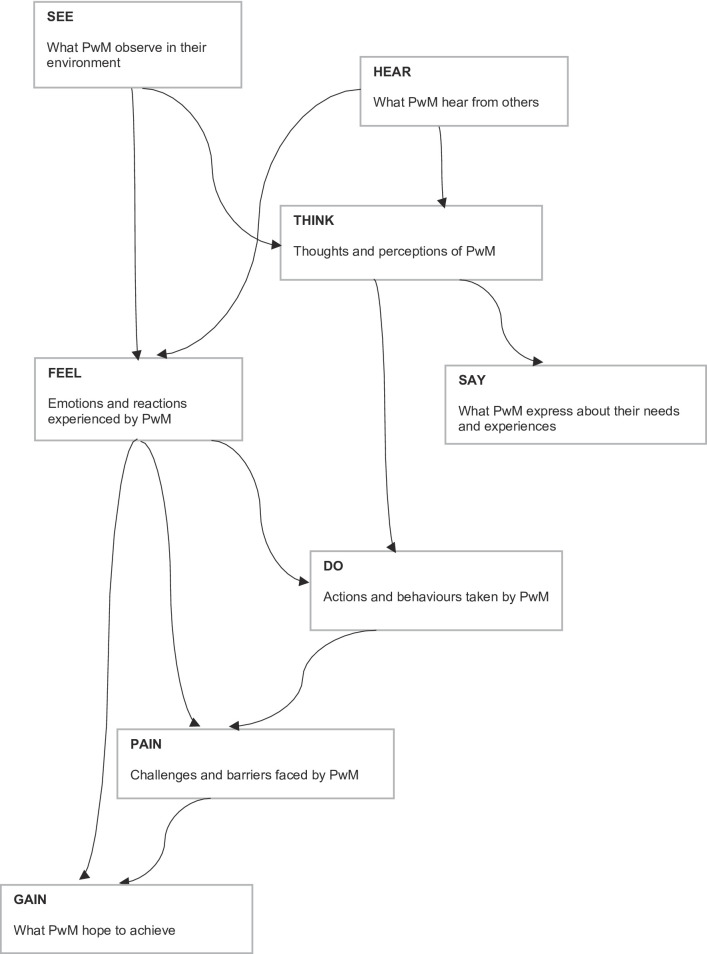
Key Dimensions of Empathy Map for PwM in HPC

We identified eight themes based on the dimensions of the empathy map. As a result of the analysis, we visualised the interrelationships among the empathy map dimensions in Fig. 1.

### Hear: What PwM Hear from Others

Healthcare providers observed that PwM often hear confusing information about their diagnosis and care. This confusion stemmed from cultural expectations and family influences. These dynamics led to disagreements within families about sharing diagnostic information or differing views on care strategies. One healthcare provider noted:

*According to the relatives*,* the patient was not supposed to learn about the diagnosis. The desire for therapy was also present*,* depending on the circumstances.* (R-252)

Differences in families’ perceptions or discussions of care can emerge in various forms. In some cases, relatives had different expectations about the treatment process or the information shared with PwM, which could create confusion for both families and healthcare providers. As one respondent summarised, “*different views on care*” (R-308), illustrating how such differences can shape communication and decision-making around care.

Significant challenges arising from “*language barriers and cultural misunderstandings*” (R-44) hindered effective communication and care. Healthcare providers observed prejudices and a lack of willingness among staff to address the specific needs of PwM. Moreover, some colleagues expressed the view that PwM should adapt to German healthcare standards rather than receive culturally sensitive care. As one healthcare provider explained:

*Prejudices and a lack of willingness on the part of many employees*,* as in other healthcare sectors*,* to engage with the special*,* very different needs of families with a migration background. It is not uncommon to hear the phrase: ‘Anyone who wants to receive care in Germany has to adapt.’* (R-125).

### See: What PwM Observe in their Environment

Healthcare providers described that PwM observed conflicts arising between SAPV teams and hospitals regarding the extent of therapy provided to them. One healthcare provider noted, “*there were frequent tensions with the SAPV team and the hospital about how far the therapy should go*” (R-197). Moreover, cultural misunderstandings among nurses, physicians, and PwM sometimes led to communication breakdowns and difficulties in delivering appropriate care. As another healthcare provider reported, “*cultural diversity sometimes resulted in nurses and doctors not properly understanding patients*,* which made collaboration difficult*” (R-69). These observations highlight the need to strengthen cultural sensitivity and build more effective communication pathways to enhance care quality and minimise conflicts.

From the perspective of healthcare providers, PwM often found themselves in environments that felt unfamiliar and culturally distant, which can create barriers to understanding and accessibility. Healthcare providers noted that even when information was available in hospitals, many PwM struggled to fully grasp the context of care due to language barriers: “*Although information is provided in the hospitals*,* it is often difficult to make sense of the context due to language barriers*” (R-102). Beyond language, PwM were seen to perceive a predominantly Christian character of some hospice facilities, reflected in symbols, colours, and spiritual services. According to the healthcare providers, these settings did not always align with the diverse religious and cultural backgrounds of PwM and could lead to feelings of exclusion or discomfort in HPC facilities. As one respondent illustrated:

*The largest outpatient and inpatient hospice facility in the region has an explicitly Christian character: ‘We also accept patients of other religious affiliations*,*’ but symbols*,* colours and attitude (memorial services in the church as the only spiritual service) of church-bound Christianity cannot be authentically reconciled.* (R-125)

The lack of cultural and linguistic representation, along with healthcare providers’ limited awareness and understanding of religious and cultural differences, further isolated PwM. Healthcare providers described how a lack of staff who could communicate in patients’ native languages or understand their cultural and religious backgrounds created barriers to effective care. In some cases, this resulted in practical and emotional challenges for both staff and PwM. One healthcare provider noted that “*the nursing staff had the impression that they were unable to fulfill their duties*” (R-155), while another reported that “*the patient was supposed to receive education*,* but a supporting carer could not be found*” (R-37). Similarly, uncertainties among volunteers and limited knowledge of different religious or cultural practices further hindered inclusive care: “*Reluctance to engage*,* uncertainties among volunteers*,* limited knowledge of the religious specifics of other religions and cultures*” (R-30). These examples show how limited representation and awareness can hinder culturally sensitive care and increase emotional strain for both staff and PwM.

PwM appeared to experience the consequences of a fragmented healthcare system, where services were disconnected and poorly coordinated. Challenges such as inadequate communication between hospitals and general practitioners and the lack of culturally tailored resources, such as interpreters, made it difficult for PwM to access appropriate care. As one healthcare provider explained, “*communication deficits with the family doctor and hospital*” (R-269) frequently disrupted continuity of care. Administrative processes became especially challenging when responsibilities were fragmented, and PwM and their families struggled to coordinate essential services. As described by another healthcare provider, “*no overarching responsibilities; everyone only focused on their specific area*,* making administrative matters particularly difficult or nearly impossible to resolve*” (R-88).

In addition to these structural issues, many PwM lacked access to clear and culturally appropriate information about available services. One healthcare provider stated that “*people with a migration background do not know about our work and we do not yet provide sufficient information in this regard*” (R-144). These gaps in communication and access made systemic fragmentation visible to PwM and shaped how they perceived the healthcare environment around them.

### Feel: Emotions and Reactions Experienced by PwM

Healthcare providers observed that PwM’s feelings of fear and anxiety made it necessary for them to approach care with heightened sensitivity. Their emotions were shaped by unfamiliarity with the healthcare system, cultural misunderstandings, and concerns about receiving inadequate care. Situations where PwM reacted negatively to discussions about death and dying, sometimes perceiving them as suggestions of euthanasia, revealed how cultural misunderstandings can exacerbate sensitive issues. As healthcare providers stated:

*‘The possibility of dying was discussed’*,* which took away the will to live and led to accusations of euthanasia.* (R-251)

… *the professionals […]*,* all of whom seemed completely overwhelmed by the situation and at times showed very little understanding and empathy.* (R-88)

According to healthcare providers’ observations, in some cases, PwM and their families struggled to accept the transition to palliative care and continued to seek active treatment despite limited medical indications. Healthcare providers also encountered PwM grappling with severe emotional distress, including suicidal thoughts and a loss of will to live. As one healthcare provider noted, “*the indication for continuing chemotherapy was borderline*,* but the patient always demanded therapy. He and his wife could not accept the palliative situation*” (R-186). Another case described, “*the patient is currently still in care*,* has lost the will to live*,* is at risk of suicide*,* and struggles with alcohol problems*” (R-142). These examples show that PwM and their families often found it hard to accept the situation.

### Think: Thoughts and Perceptions of PwM

Language limitations, as observed by healthcare providers, affected both spoken communication and PwM’s ability to access essential details about palliative care. Since the websites of HPC facilities and other information resources were almost exclusively available in German, PwM felt excluded from understanding and making informed decisions about their care. As one healthcare provider noted, “*hospice websites are almost exclusively in German. Flyers and other information material either*” (R-35). This lack of accessible information also contributed to misunderstandings and misconceptions about palliative care. As another respondent explained, “*the level of information about care is limited. It is not known what palliative medicine is. Cultural barriers are conceivable*,* as it is assumed that palliative medicine is euthanasia*” (R-78). Moreover, prejudices within families further reinforced these perceptions, with one healthcare provider observing, “*palliative medicine is seen as giving up on treatment and opportunities; it takes away all hope*” (R-189).

Beyond issues of information access and understanding, some PwM also questioned the fairness of the care they received. Healthcare providers noted that some PwM perceived inequalities in the healthcare system and questioned whether they were receiving the same quality of care as the local population. Feelings of mistrust emerged, especially when PwM believed that not all possible treatments were being offered to them because of their migration background. As one healthcare provider stated, “*initially*,* there were significant reservations and pronounced mistrust*,* with a suspicion that not everything was being done for him*” (R-279). Language barriers further deepened these perceptions, particularly among those who did not speak German or English, as one respondent observed: “*Non-German or non-English speaking citizens may not receive adequate care*” (R-237).

### Say: What PwM Express about their Needs and Experiences

According to healthcare providers, PwM expressed frustration with language barriers that limited their ability to understand care options and communicate effectively about treatment. Healthcare providers reported that PwM emphasised the need for information about HPC in their native languages to better navigate the system. As one respondent summarised, “*mainly communication problems*,* no discussion about therapy possible*” (R-319). Another healthcare provider added, “*lack of information about care options in the mother tongue*” (R-219). PwM also expressed broader concerns that combined linguistic, financial, and cultural challenges:

*[They say:] ‘I don’t even know what a hospice is.’ Financial concerns: ‘I can’t afford the hospice.’ And: ‘Can I live my faith in a Christian facility? They don’t speak my language*,* they don’t know how I feel*,* and I’m also different.’* (R-243).

It was observed by healthcare providers that PwM conveyed deep concerns about preserving their cultural and religious practices in care settings. They expressed the need to maintain faith-based rituals and traditions, even in environments that felt unfamiliar or lacked cultural awareness. PwM also stressed how family support was crucial for sustaining emotional well-being and preserving cultural identity. As one healthcare provider summarised, “*access barriers arising from social and religious background*” (R-6). Another healthcare provider reflected the emotional burden of this challenge: “*Culture happens in the family; what to do if there is no family here?*” (R-153).

### Do: Actions and Behaviours Taken by PwM

Many PwM, as shared by healthcare providers, only considered palliative care when their condition became severe. This delay was linked to language barriers, limited awareness of available services, and fears of discrimination. In some cases, families had difficulty managing care at home, resulting in repeated hospitalisations. As one healthcare provider noted:

*The relatives were not entirely satisfied with the palliative care*,* and the patient had to be taken to the hospital repeatedly because the children could not cope with the situation.* (R-169)

Some PwM also resisted changes to their treatment plans, as they requested maximal therapy based on their experiences in their country of origin, which made it even more difficult to provide timely and effective care. As another healthcare provider stated, “*rejection of changes in treatment goals*,* maximal therapy is sometimes requested (depending on experiences in the home country)*” (R-46).

A great deal of reliance on family, community, or religious networks for support throughout their care journey was commonly observed. In many cases, these networks played a central role in organising assistance and compensating for gaps in formal care structures. PwM generally turned to religious communities or non-family supporters when professional or institutional support felt insufficient. As one healthcare provider described:

*The patient was completely dependent on the support of her non-family network*,* which she could not or would not fully acknowledge. As a result*,* supporters outside the hospice service reached their limits. The patient demanded a lot of support from the religious community.* (R-105)

Family members played an active role as advocates, interpreters, and mediators between PwM and healthcare providers. They often acted as the main point of contact for PwM to ensure their preferences were communicated. As one healthcare provider explained, “*son as translator and primary contact person (as desired by the son and the patient)*” (R-266). However, healthcare providers also encountered situations where families exercised strong control over communication and decision-making, at times limiting the involvement of PwM in their own care. One healthcare provider noted:

*The family of the patient demanded that the truth not be disclosed. The behaviour of the family was very overbearing and controlling toward the mother*,* who was not to be included in decision-making.* (R-269)

### Pain: Challenges and Barriers Faced by PwM

Healthcare providers observed that language barriers significantly hindered the understanding of HPC services by PwM. Even simple conversations about treatment or care options could become sources of stress and misunderstanding. In some cases, PwM were unsure what hospice care meant compared with hospital care, as one healthcare provider explained: “*Language barrier*,* which also exacerbated the problem that there was no understanding of what a hospice is compared to a hospital*” (R-307). Clear communication was also not always guaranteed when both sides shared a migration background, as another healthcare provider noted: “*The hospital doctor also had a migration background*,* which made clear communication somewhat difficult*” (R-215).

Beyond language barriers, experiences of discrimination and stigma further complicated the situation for PwM and their families. Counselling and support were often inadequate for non-German-speaking families, particularly when interpreter services were needed, which caused delays in both information exchange and access to care. One healthcare provider described how “… *families who do not speak German are often offered fewer consultations*,* because an interpreter is required. This sometimes delays the provision of information and connection to services*” (R-19). Discriminatory attitudes also appeared in care environments, such as “*Christian sponsors who do not want to visit people of other religions and immigrant backgrounds*” (R-44). Misunderstandings were sometimes interpreted as a lack of intelligence, as another healthcare provider stated: “*Don’t understand is often equated with stupid*” (R-194). Certain perceptions reinforced exclusion, as another provider explained: “*Prejudices that arise early among the providers*,* such as ‘They’ll do it themselves anyway; they don’t want anyone from outside*’” (R-269). Such biases fostered feelings of rejection and deepened mistrust among PwM toward healthcare systems.

### Gain: What PwM Hope to Achieve

According to healthcare providers, PwM placed great importance on receiving care that maintained their dignity and respected their cultural and religious identities. Many PwM sought care environments where their traditions and beliefs around illness and dying were understood and integrated into care practices. To meet these expectations, healthcare providers emphasised the need for more comprehensive training that would strengthen their cultural competence and sensitivity. As one healthcare provider explained, “*acquired knowledge of culture-specific rites*,* beliefs*,* and customs to communicate and act in a culturally sensitive manner*” (R-167). Others pointed out the importance of “*training on world religions and culture-specific differences in coping with illness and dying*” (R-84) and “*intercultural training on the topics of caregiving*,* family culture*,* and religious rites in Islam*” (R-143).

Building and maintaining mutual trust between PwM and healthcare providers was essential for effective and compassionate care. Trust was strengthened when healthcare providers showed cultural understanding, maintained ongoing communication with families, and collaborated with community and religious networks. As one healthcare provider suggested, “*employing volunteers with a migration background*” (R-195) could help PwM feel more understood and comfortable within the care environment. Another healthcare provider described the importance of “*advice for the family at all times*,* phone calls with hospital doctors*,* organisation of a home palliative care service*” (R-47), illustrating how consistent communication supports trust. Observing cultural and faith-based needs was also a key factor, as respondents mentioned the “*observance of religious and ethnic rules*” (R-92) and the importance of “*contacting communities*,* religious groups*” (R-243). Together, these efforts enabled PwM to feel respected, valued, and supported in the palliative care process.

## Discussion

Our study provides a clear overview of the experiences, challenges, and needs of PwM in HPC in Germany, as reported by healthcare providers. Using Dave Gray’s [20] empathy map, we identified key themes related to the complexities of delivering culturally sensitive care. These barriers included language difficulties, cultural misunderstandings, and challenges in building trust. Our findings are consistent with existing literature, which stresses the critical role of cultural competence and language barriers in addressing disparities in HPC access and quality for minority groups [3, 9, 11, 27].

Previous studies show that language barriers remain among the most significant obstacles to effective communication and care delivery, leading to frustration, delays, and suboptimal outcomes [11, 12, 28]. Our study adds new insights by revealing that not only the lack of interpreters but also the absence of multilingual materials, such as brochures and websites, worsens the exclusion of PwM from essential information. These results are consistent with the broader literature on the necessity of culturally adapted communication strategies [1]. While Cairns et al. [19] demonstrated the value of empathy maps as a communication and learning tool for improving understanding between provider-beneficiary, our study offers a different contribution by applying the empathy map as an analytical framework to interpret healthcare providers’ experiences and to explore their perspectives on care delivery. This distinction approach illustrates how the same conceptual tool can serve both educational and analytical purposes, effectively linking practical care experiences to the development of future empathy-focused training.

Moreover, the cultural insensitivity we observed, including prejudices and reluctance to adapt to cultural practices, reflects the findings of Jansky et al. [1] and Boucher and Johnson [17], emphasising the need for culturally tailored training. Our findings suggest that training programs should include modules on cultural norms, religious practices, and family roles, as well as strategies for managing biases and fostering inclusive environments [5–7]. Efforts to address language barriers should go beyond interpreter services and cover the development of multilingual resources [29, 30]. Providing information in different languages and training healthcare staff in basic cross-cultural communication skills can empower PwM to make informed decisions about their treatment and care [31]. Digital tools, such as interactive empathy maps and patient portals, can further support better communication and engagement [32, 33].

Recognising the central role of family and community in the care of PwM, healthcare providers should adopt strategies to constructively involve these networks. Family-centred care models, which balance the patient’s autonomy with the family’s cultural and emotional needs, can help mitigate conflicts and enhance collaboration [14, 34]. Partnerships with religious and community representatives may also foster trust and provide culturally appropriate support [35]. Our study further highlights how administrative inefficiencies and cross-border issues disproportionately affect PwM [36] and the demand for policy-level interventions to improve care pathways and access.

### Strengths and Limitations

One of the strengths of our study is its methodological rigour, particularly the innovative use of the empathy map to structure and analyse diverse healthcare provider perspectives. While the focus on Germany may limit the generalisability of findings to other contexts, the inclusion of multiple healthcare settings enhances the study’s relevance and applicability within the German HPC system. Several limitations should also be acknowledged. First, the reliance on self-reported data may introduce bias, as healthcare providers might underreport or overemphasise certain challenges. Second, our study relies solely on healthcare providers’ views, which may not provide a complete picture of PwM experiences in palliative care. Future research should directly include PwM perspectives and examine the long-term effects of culturally sensitive HPC on their quality of life and satisfaction. It could further explore integrating empathy maps as both an educational tool and an analytical framework, building on our approach to combine practical healthcare experiences with empathy training, and thereby enhancing culturally sensitive palliative care for PwM.

### New Contribution to the Literature

Empathy mapping, originally developed for user experience design, was adapted for our research context to visualise and articulate healthcare providers’ perspectives. Our study contributes to the current knowledge on culturally sensitive HPC for minority populations. Unlike previous research focused on patients’ perspectives, our research offers a unique perspective on the challenges healthcare providers face in delivering culturally sensitive care. Moreover, by employing empathy mapping as an analytical framework, our study introduces an innovative methodological perspective to palliative care research.

## Data Availability

No datasets were generated or analysed during the current study.
